# Mechanisms of Antimicrobial Resistance in ESKAPE Pathogens

**DOI:** 10.1155/2016/2475067

**Published:** 2016-05-05

**Authors:** Sirijan Santajit, Nitaya Indrawattana

**Affiliations:** Department of Microbiology and Immunology, Faculty of Tropical Medicine, Mahidol University, Bangkok 10400, Thailand

## Abstract

The ESKAPE pathogens (*Enterococcus faecium*,* Staphylococcus aureus*,* Klebsiella pneumoniae*,* Acinetobacter baumannii*,* Pseudomonas aeruginosa*, and* Enterobacter* species) are the leading cause of nosocomial infections throughout the world. Most of them are multidrug resistant isolates, which is one of the greatest challenges in clinical practice. Multidrug resistance is amongst the top three threats to global public health and is usually caused by excessive drug usage or prescription, inappropriate use of antimicrobials, and substandard pharmaceuticals. Understanding the resistance mechanisms of these bacteria is crucial for the development of novel antimicrobial agents or other alternative tools to combat these public health challenges. Greater mechanistic understanding would also aid in the prediction of underlying or even unknown mechanisms of resistance, which could be applied to other emerging multidrug resistant pathogens. In this review, we summarize the known antimicrobial resistance mechanisms of ESKAPE pathogens.

## 1. Introduction

Nosocomial infections are caused by a variety of organisms, including bacteria, fungi, viruses, parasites, and other agents. Infections can be derived from exogenous or endogenous sources and are transferred by either direct or indirect contact between patients, healthcare workers, contaminated objects, visitors, or even various environmental sources. A survey of hospital-acquired infections (HAI) in the United States in 2011 reported a total of about 722,000 reported cases, with 75,000 deaths associated with nosocomial infections [1]. A second study conducted in 2002 estimated that when taking into account all types of bacterial infections, approximately 1.7 million patients suffered from HAIs, which contributed to the deaths of 99,000 patients per year [2].

The growing numbers of antimicrobial-resistant pathogens, which are increasingly associated with nosocomial infection, place a significant burden on healthcare systems and have important global economic costs. Effects include high mortality and morbidity rates, increased treatment costs, diagnostic uncertainties, and lack of trust in orthodox medicine. Recent reports using data from hospital-based surveillance studies as well as from the Infectious Diseases Society of America have begun to refer to a group of nosocomial pathogens as “ESKAPE pathogens” [3, 4]. ESKAPE is an acronym for the group of bacteria, encompassing both Gram-positive and Gram-negative species, made up of* Enterococcus faecium*,* Staphylococcus aureus*,* Klebsiella pneumoniae*,* Acinetobacter baumannii*,* Pseudomonas aeruginosa*, and* Enterobacter* species. These bacteria are common causes of life-threatening nosocomial infections amongst critically ill and immunocompromised individuals and are characterized by potential drug resistance mechanisms [5].

## 2. Antimicrobial Resistance Mechanisms of ESKAPE Pathogens

Antimicrobial resistance genes may be carried on the bacterial chromosome, plasmid, or transposons [6]. Mechanisms of drug resistance fall into several broad categories, including drug inactivation/alteration, modification of drug binding sites/targets, changes in cell permeability resulting in reduced intracellular drug accumulation, and biofilm formation [7–9].

### 2.1. Drug Inactivation or Alteration

Many bacteria produce enzymes that irreversibly modify and inactivate the antibiotics, such as *β*-lactamases, aminoglycoside-modifying enzymes, or chloramphenicol acetyltransferases. One of the well-characterized enzymes is *β*-lactamases. They are highly prevalent and act by hydrolyzing the *β*-lactam ring which is present in all *β*-lactams; thus, all penicillins, cephalosporins, monobactams, and carbapenems are essential to their activity [10]. *β*-lactamases are classified using two main classification systems: the Ambler scheme (molecular classification) and the Bush-Jacoby-Medeiros system, which classifies the most clinically important *β*-lactamases as those produced by Gram-negative bacteria [4]. Ambler class A enzymes consist of penicillinase, cephalosporinase, broad-spectrum *β*-lactamases, extended-spectrum *β*-lactamases (ESBLs), and carbapenemases. They can inactivate penicillins (except temocillin), third-generation oxyimino-cephalosporins (e.g., ceftazidime, cefotaxime, and ceftriaxone), aztreonam, cefamandole, cefoperazone, and methoxy-cephalosporins (e.g., cephamycins and carbapenems). Class A enzymes can also be inhibited by *β*-lactamase inhibitors, such as clavulanic acid, sulbactam, or tazobactam [5, 6].

The Ambler class A group contains a number of significant enzymes including ESBLs (mainly TEM, SHV, and CTX-M type) and KPCs. TEM-type enzymes were first identified in 1965 in* Escherichia coli* from Greek patients, with TEM taken from a patient's name, Temoniera. TEM-1 is widespread not only amongst bacteria of the family Enterobacteriaceae (e.g.,* K. pneumoniae*,* Enterobacter* spp.), but also in nonfermentative bacteria such as* P. aeruginosa*. Currently, TEM enzymes are the most common group in* E. coli*. Amongst sulfhydryl variable (SHV) enzymes, SHV-1 is the most clinically relevant and represents the most common* K. pneumoniae* [11]. The genes coding for TEM and SHV enzymes have quite high mutation rates, resulting in a high level of diversity in enzyme types and thus increasing the scope of antibiotic resistance. CTX-Ms have been identified in ESKAPE pathogens including* K. pneumoniae*,* A. baumannii*,* P. aeruginosa*, and* Enterobacter* species. Some of the highest prevalence and significant clinical impact are associated with the extended-spectrum *β*-lactamases in* K. pneumoniae* [12, 13]. Carbapenemases are also prevalent in clinical bacterial isolates such as* K. pneumonia* such as KPC-1 that results in resistance to imipenem, meropenem, amoxicillin/clavulanate, piperacillin/tazobactam, ceftazidime, aztreonam, and ceftriaxone [14].

Ambler class B enzymes, or group 3 enzymes as classified by the Bush-Jacoby system (Table 1), include metallo-*β*-lactamases (MBLs), which require Zn^2+^ as a cofactor. Bacteria that produce these enzymes show resistance to all *β*-lactams, including penicillins, cephalosporins, carbapenems, and *β*-lactamase inhibitors, except aztreonam. Genes encoding MBLs are found on plasmids; hence, they are easily transmitted to other microorganisms. The most common metallo-*β*-lactamases (MBLs) are imipenemase metallo-*β*-lactamases (IMP), Verona integron encoded metallo-*β*-lactamases (VIM), and the newly described New Delhi metallo-beta-lactamase-1 (NDM-1) enzymes [5, 15]. IMP-type MBLs have mainly been found in* P. aeruginosa*,* K. pneumoniae*,* A. baumannii*, and* Enterobacter cloacae*, whereas VIM-type enzymes have been detected mostly in* P. aeruginosa* and* A. baumannii*. NDM-1-type enzymes have been isolated from* K. pneumoniae* and* E. cloacae* [5, 16].

The Ambler class C group consists of several important enzymes, including penicillinase and cephalosporinase, such as AmpC *β*-lactamase, which results in low level resistance to narrow-spectrum cephalosporin drugs. Chromosomally encoded AmpC are usually identified in* P. aeruginosa* and bacteria in the Enterobacteriaceae family such as* Enterobacter* species where their production is typically very low level and does not elicit any clinically relevant resistance but can be inducible during drug therapy. Nevertheless, the acquisition of transmissible plasmids from other bacteria can lead to the overproduction of AmpC *β*-lactamase in some organisms ordinarily lacking the gene encoding for chromosomal AmpC, for example,* K. pneumonia* [17]. The AmpC *β*-lactamases inactivate aztreonam, all penicillin, and most cephalosporins and are not susceptible to inhibition by most *β*-lactamases inhibitors except avibactam, a new non-*β*-lactam *β*-lactamase inhibitor antibiotic [4].

Ambler class D consists of a variety of enzymes, such as oxacillin hydrolyzing enzymes (OXA). The most common members of this class, such as OXA-11, OXA-14, and OXA-16, demonstrate ESBL properties and are normally found in* P. aeruginosa* [11, 18, 19]. OXA enzymes are classified as group 2d following the Bush-Jacoby scheme, and almost all of these enzymes, except OXA-18, are resistant to *β*-lactamase inhibitors [20]. Furthermore, OXA-type carbapenemases are commonly found in* Acinetobacter* spp. Specific* A. baumannii* carbapenem-hydrolyzing OXA enzymes, which have low catalytic efficiency, together with porin deletion and other antibiotic resistance mechanisms, can cause high resistance to carbapenems [21].

### 2.2. Modification of Drug Binding Sites

Some resistant bacteria avoid recognition by antimicrobial agents by modifying their target sites. The mutation of gene encoding for penicillin-binding proteins (PBPs), which are enzymes typically anchored on the cytoplasmic membrane of the bacterial cell wall and function in assembly and control of the latter stages of the cell wall building, results in the expression of unique penicillin-binding proteins, for example, the expression of a unique PBP2a in* S. aureus*, which is the most dominant PBP in the MRSA cell compared to the native PBPs (PBP1–4) [22]. PBP2a has low affinity for all *β*-lactam antibiotics and acts as a substitute for the other PBPs, thus enabling the survival of* S. aureus* in the presence of high concentrations of *β*-lactam drugs including methicillin acting on cell wall biosynthesis [23]. Bacterial cell wall synthesis in methicillin-resistant Gram-positive organisms can be inhibited by glycopeptides, which target acyl-D-alanyl-D-alanine (acyl D-Ala-D-Ala) residues of peptidoglycan precursors. However, by changing the peptidoglycan cross-link target (D-Ala-D-Ala to D-Ala-D-Lac or D-Ala-D-Ser), encoded by a complex gene cluster (*Van-*A,* Van-*B,* Van-*D,* Van-*C,* Van-*E, and* Van-*G),* E. faecium* and* E. faecalis* can increase their resistance to glycopeptides in current clinical use (vancomycin and teicoplanin) [6].

### 2.3. Reduced Intracellular Drug Accumulation

The balance of antibiotic uptake and elimination determines the susceptibility of bacteria to a particular drug. Thus, reducing the amount of antibiotic able to pass through the bacterial cell membrane is one strategy used by bacteria to develop antibiotic resistance. Mechanisms by which bacteria achieve this include the occurrence of diminished protein channels on the bacterial outer membrane to decrease drug entry and/or the presence of efflux pumps to decrease the amount of drug accumulated within the cells.

#### 2.3.1. Porin Loss

The outer membranes of Gram-negative bacteria contain proteins called porins that form channels that allow the passage of many hydrophilic substances, including antibiotics. A reduction in the amount of* P. aeruginosa* porin protein OprD results in decreased drug influx into the cell, allowing the bacterium to develop resistance to imipenem [24]. Loss of a 29-kDa outer membrane protein (OMP) in other Gram-negative bacteria, such as* A. baumannii*, allows them to become insensitive to imipenem and meropenem drugs. Multiple-drug resistant* K. pneumoniae* strains also exhibit resistant/reduced susceptibility to *β*-lactams (such as cephalosporins and carbapenems) by the loss of outer membrane proteins known as OmpK35 and OmpK36 together with the production of resistance enzymes, including AmpC *β*-lactamase and new-generation carbapenemase A, KPC [21].

#### 2.3.2. Efflux Pumps

To increase the removal of antibiotics from the intracellular compartment (or the intermembrane space in Gram-negative bacteria), some bacteria contain membrane proteins that function as exporters, called efflux pumps, for certain antimicrobial agents. These pumps expel the drug from the cell at a high rate, meaning that the drug concentrations are never sufficiently high to elicit an antibacterial effect. Most efflux pumps are multidrug transporters that efficiently pump a wide range of antibiotics, contributing to multidrug resistance. Up to date, there are five super families of efflux pumps that have been described. These include the ATP-binding cassette (ABC) family, the small multidrug resistance family, the major facilitator super family, the resistance-nodulation-division (RND) family, and the multidrug and toxic compound extrusion family [25].

The most common type of efflux pump in Gram-negative bacteria is the polyselective efflux pump, belonging to the RND superfamily, which plays a key role in the multidrug resistance (MDR) bacterial phenotype. This type of pump expels a variety of antibiotics and structurally unrelated molecules, such as dyes and bile salts, but also detergents and biocides that are frequently used in medical practice [26]. AcrAB-TolC and MexAB-OprM are multidrug efflux pumps typically belonging to the RND superfamily. They are usually chromosomally encoded. These two efflux pumps are essential for bacterial survival, particularly in the presence of toxic agents.* P. aeruginosa* contains a large number of efflux pumps, with four potent RND-type multidrug resistance efflux pumps (Mex) capable of eliminating toxic compounds from the periplasm and cytoplasm. Two of these efflux pumps, MexAB-OprM and MexCD-OprJ, are responsible for resistance to at least three main classes of antibiotics, namely, carbapenems, fluoroquinolones, and aminoglycosides [27, 28]. Studies have shown that overexpression of MexXY-OprM from* P. aeruginosa* results in resistance to aminoglycosides, fluoroquinolones, and specific antipseudomonal cephalosporins. Furthermore, many clinical* P. aeruginosa* isolates also express MexCD-OprJ and MexEF-OprN.

An increase in the prevalence of strains overproducing these efflux pumps has also been reported in* Enterobacter aerogenes* and* K. pneumoniae* clinical isolates. Overexpression of the AcrAB efflux pump, together with decreased expression of porins, is characteristic of imipenem-resistant* E. aerogenes* MDR strains. In these bacteria, the efflux pump also ejects other unrelated antibiotics, such as fluoroquinolones, tetracycline, and chloramphenicol.* A. baumannii* isolates can also demonstrate a MDR phenotype through the presence and overexpression of RND efflux pump AdeABC. This pump is associated with resistance to a broad range of antibiotics, including fluoroquinolones, *β*-lactams, tetracyclines (including tigecycline), macrolides/lincosamides, chloramphenicol, and aminoglycosides. Like* P. aeruginosa*,* A. baumannii* porins also show very low permeability. The RND-type efflux pumps AdeABC, AdeDE, AdeFGH, and AdeIJK play a role in resistance to aminoglycosides, fluoroquinolones, erythromycin, tetracycline, and chloramphenicol in all bacterial species reported to date. Finally, the synergistic effect of multidrug efflux pumps and the outer membrane barrier is important for resistance to many agents. For example, the main porin expressed by* P. aeruginosa* is OprF, which has much lower permeability than* E. coli* OmpF, making the efflux pump activity more effective in resistant strains [26].

### 2.4. Biofilm Formation

Biofilms are complex microbial communities living as a thin layer on biotic or abiotic surfaces, implanted in a matrix of extracellular polymeric substances created by the biofilms themselves. Microorganisms within the biofilm can interact with each other, as well as the environment. The major component of the matrix is secreted extracellular polymeric substances, mainly consisting of polysaccharides, proteins, lipids, and extracellular DNA from the microbes [29]. There are three key steps for biofilm formation. The first step is adhesion, which occurs as cells reach a surface and anchor to the site. The second step is growth and maturation, which happens as the microbes begin to generate the exopolysaccharide that establishes the matrix and then mature from microcolonies to multilayered cell bunches. The final step is detachment, which can be divided into two types: active and passive. Active detachment is initiated by bacteria themselves, for example, by quorum sensing and enzymatic degradation of the biofilm matrix. In contrast, passive detachment is caused by external forces, such as fluid shear, scraping, and human intervention [30].

It could be argued that the main causes of antimicrobial resistance are not classical drug resistance mechanisms, that is, efflux pumps, target site modification, or enzymatic degradation. It is likely that the matrix of biofilms provides a mechanical and biochemical shield that provides the conditions needed to attenuate the activity of the drugs (e.g., low O_2_, low pH, high CO_2_, and low water availability). Under these conditions it is difficult to eliminate bacteria using conventional antibiotics. Moreover, when the bacteria experience nutrient scarcity, they could become tolerant to antibiotics. This may explain the apparent greater antibiotic resistance of cells in the deep layers of a biofilm (bacteria extracted from the biofilms and grown in broth recover their full susceptibility, indicating that the resistance is phenotypic and not genotypic) [31]. The most common pathogens found in biofilms in a healthcare setting are* S. aureus*,* P. aeruginosa*,* A. baumannii*, and* K. pneumonia* [32].

## 3. Antibiotic Resistance in ESKAPE Pathogens

### 3.1. *Enterococcus faecium*

*Enterococcus* species were formerly classified as part of the genus* Streptococcus*. They are Gram-positive facultative anaerobes, which are often found in pairs or chains. Their normal habitat is the gut of humans and animals. There are more than 20* Enterococcus* species, but* Enterococcus faecium* and* Enterococcus faecalis* are the most clinically relevant. Most* Enterococcus* infections are endogenously acquired, but cross-infection may occur in hospitalized patients [33]. Over the past decade, some reports have revealed a rise in ampicillin- and vancomycin-resistant enterococcal infections in healthcare facilities. For instance, in Netherlands, the average number of invasive ampicillin-resistant enterococcal infections in university hospitals escalated from approximately 10 infections in 1999 to 50 infections in 2005 per hospital [34]. Rates of antimicrobial resistance amongst enterococci are particularly concerning, especially the incidence of vancomycin-resistant* Enterococcus* (VRE), which is mainly associated with* E. faecium*. VRE emerged in North America during the late 1980s, with 61% of* E. faecium* isolates estimated to be vancomycin resistant by 2002. While the incidence of VRE is much lower in European countries, including Ireland and the United Kingdom, a survey by the British Society for Antimicrobial Chemotherapy from 2001 to 2006 found that the incidence of VRE in Europe had risen from approximately 20% to more than 30% [35, 36]. Despite the high global rates of VRE, there is some geographical variation. There are six types of VRE (Van-A–E and Van-G), with* van-A* being the most prevalent and showing the highest levels of resistance to all glycopeptide antibiotics [37]. In 2011, Galloway-Pena and her colleagues demonstrated two diverse clades of* E. faecium* which differ genetically. Clade A clinical isolates were found to associate predominantly with hospitals, whereas clade B isolates were associated with community origin. Both clades express low-affinity penicillin-binding proteins (called PBP5) which bind weakly to *β*-lactam drugs. In addition, clade A has acquired several virulence determinants and resistance genes from the presence of insertion sequence 16 (IS16) and a gene encoding the ampicillin-resistant PBP5 (pbp5R) while clade B has been shown to have a gene encoding for ampicillin-sensitive PBP5 (pbp5S) [38].

### 3.2. *Staphylococcus aureus*

*S. aureus* is a Gram-positive coccal bacterium, with cells arranged in characteristic grape-like clusters. With nonfastidious growth requirements,* S. aureus* is part of the normal skin flora, especially of the nose and perineum of humans and animals. Carriage rates are high in the general population, and transmission can occur by direct contact or airborne routes. Traditionally, infections caused by* Staphylococcus* species have responded well to penicillin treatment; however, excessive use of these antibiotics led to the emergence of *β*-lactamase-producing* Staphylococcus* isolates in 1948, with 65–85% of staphylococcal clinical isolates now also resistant to penicillin G. In two decades, the incident of *β*-lactamase-producing* Staphylococcus* species increased more than 80% in both community and hospital associated infections as reported by Bodonakik et al., 1984; Appelbaum and Brown, 2007; and Wu et al., 2010 [39–42]. Reports of methicillin-resistant* Staphylococcus aureus* (MRSA) emerged in the 1960s, and currently, MRSA isolates are estimated to account for 25% of* S. aureus* isolates, with a prevalence of up to 50% or more in some areas. Researchers from the Prince of Songkhla Hospital, Prasat Neurological Institute, and Hospital for Tropical Diseases, Thailand, studied the prevalence of methicillin resistance amongst 92 clinical* S. aureus* isolates. Of these isolates, 60.9% were MRSA, and all were sensitive to vancomycin [43]. Tackling the problem of MRSA is a top priority for public health systems worldwide, with much current research focused on future intervention strategies.

In most cases, glycopeptide antibiotics, for example, vancomycin and teicoplanin, are used as first-line antibiotics for treatment of MRSA infections. However, the selective pressure of these antibiotics has induced some strains to become intermediate-susceptible to vancomycin* in vitro*, with cases of clinical vancomycin-intermediate and vancomycin-resistant* S. aureus* (VISA and VRSA, resp.) becoming more common [44]. Unfortunately, most VISA isolates are also less susceptible to teicoplanin, with the term glycopeptide-intermediate* S. aureus* used to identify these isolates. VISA was first reported in Japan in the mid-1990s, and the strains have now emerged in other countries across Asia, the USA, and Europe. VRSA is of particular concern because of the interspecies exchange of genetic resistance genes from VRE. VRSA isolates contain both the* van-A* and* mec-A *resistance determinants of VRE and MRSA, which result in resistance to multiple drugs, including methicillin and vancomycin [45].

### 3.3. *Klebsiella pneumonia*

*K. pneumoniae* is a member of the family Enterobacteriaceae. It is a nonfastidious, Gram-negative bacillus, which is usually encapsulated. Species of the genus* Klebsiella* are the bacterial pathogens most often found associated with infections in healthcare settings and infections may be endogenous or acquired through direct contact with an infected host. In recent years, many* K. pneumoniae* strains have acquired a massive variety of *β*-lactamase enzymes, which can destroy the chemical structure of *β*-lactam antibiotics such as penicillins, cephalosporins, and carbapenems. Because carbapenems are conventionally used to treat persistent infections caused by Gram-negative bacteria, the increasing prevalence of carbapenem-resistant* K. pneumoniae* (CRKP), with resistance encoded by bla_KPC_, presents a significant challenge for physicians [46, 47]. In addition, the emergence of the* K. pneumoniae* super enzyme, known as NDM-1 and encoded by bla_NDM-1_, has increased the proportion of carbapenem-resistant* K. pneumoniae* isolates and may pose a threat to other antibiotics such as *β*-lactams, aminoglycosides, and fluoroquinolones [15, 16]. Even if several intensive infection control practices are used, outbreaks of carbapenemase-mediated multidrug resistant (MDR) strains are only reduced and cannot be completely eradicated. An effective treatment is therefore needed to overcome these pathogens.

### 3.4. *Acinetobacter baumannii*

*Acinetobacter* species are widely distributed in the environment and readily contaminate the hospital environment. The most important human pathogen is* A. baumannii*, which has a relatively long survival time on human hands, which can lead to high rates of cross contamination in nosocomial infections [48].* A. baumannii* is a nonfermentative Gram-negative coccobacillus and causes infections at a variety of sites, including the respiratory and urinary tracts. Strains are frequently antibiotic resistant, which is a particular problem in surgical wards and intensive care units [49]. Recently, the emergence of carbapenemase-producing* A. baumannii* strains carrying imipenem metallo-*β*-lactamases, encoded by bla_IMP_, and oxacillinase serine *β*-lactamases, encoded by* bla*_OXA_, has been reported. These strains show resistance to both colistin and imipenem, and the combination of resistance genes makes them capable of evading the action of most traditional antibiotic compounds [50, 51].

### 3.5. *Pseudomonas aeruginosa*

*P. aeruginosa* is a Gram-negative, rod-shaped, facultative anaerobe that is part of the normal gut flora. Carriage rates are fairly low in the general population but are higher in hospital inpatients, especially immunocompromised hosts. Patients become infected through an exogenous source, such as by direct/indirect contact with the environment, but endogenous sources are also possible. Many* P. aeruginosa* strains show an intrinsic reduced susceptibility to several antibacterial agents, as well as a propensity to develop resistance during therapy especially in carbapenem-resistant (chiefly imipenem) strains. The most common mechanism of imipenem resistance in* P. aeruginosa* is a combination of chromosomal AmpC production and porin change. Indeed, low level of AmpC enzymes production does not result in high-level carbapenem resistance due to their low potential to hydrolyze carbapenem drugs but their overproduction together with reduced outer membrane porin permeability and/or efflux pump overexpression contribute to high-level carbapenem resistance in this pathogen [24, 33].* P. aeruginosa* also produces ESBLs and can harbor other antibiotic resistance enzymes such as* K. pneumoniae* carbapenemases (KPC), VIM encoded by bla_VIM_, and imipenem metallo-*β*-lactamases. The combination of these enzymes leads to high rates of carbapenem resistance amongst* P. aeruginosa* isolates and also to the emergence of fluoroquinolone-resistant strains as the corresponding mechanisms of resistance may be carried by the same plasmid [46, 52]. The continuous increase of MDR isolates presents a complicated situation for antimicrobial therapy; however, colistin is still effective in most cases [51].

### 3.6. *Enterobacter spp.*

*Enterobacter* species are nonfastidious Gram-negative rods that are sometimes encapsulated. They can cause opportunistic infections in immunocompromised, usually hospitalized, patients and contain a wide range of antibiotic resistance mechanisms. Many* Enterobacter* strains contain ESBLs and carbapenemases, including VIM, OXA, metallo-*β*-lactamase-1, and KPC [53]. Furthermore, stable derepression of the AmpC *β*-lactamases that can be expressed at high levels by mutation in this bacterial group is important also. These MDR strains are resistant to almost all available antimicrobial drugs, except tigecycline and colistin [51].

In conclusion, nosocomial ESKAPE bacteria represent paradigms of resistance, pathogenesis, and disease transmission. There are a range of antimicrobial resistance mechanisms used by the nosocomial ESKAPE pathogens, including enzymatic inactivation, modification of drug targets, changing cell permeability through porin loss or increase in expression of efflux pumps, and mechanical protection provided by biofilm formation. Antimicrobial resistance in these pathogens is a major menace to public health systems worldwide and seems likely to increase in the near future as resistance profiles change. This results in the dearth of potential therapeutic agents in the pipeline that causes real concerns but should trigger research and development of new antibiotics or new approaches to control the infections they cause. In this context, there are current research efforts which are focused on the introduction of new therapeutic schemes to circumvent these pathogens, including antivirulence strategies, bacteriophage therapy, probiotics, therapeutic antibodies, synthetic inhibitors specific to resistance enzymes or bacterial efflux pumps, and inhibition of biofilm formation. These novel tools provide hope for prevention and treatment of infectious diseases caused by these ESKAPE organisms.

## Figures and Tables

**Table 1:** Categorization of bacterial *β*-lactamase enzymes by the Bush-Jacoby and Ambler systems.

Classification		Antimicrobial agents	Enzymes	Description	ESKAPE pathogens
Bush-Jacoby	Ambler				
1	C	Narrow and extended-spectrum cephalosporins, including cephamycins	ACT-1, FOX-1, MIR-1, CMY	Cephalosporinases not inactivated by clavulanic acid	*Enterobacter *spp.
2a	A	Penicillins	PC1	Penicillinases inactivated by clavulanic acid	Enterobacteriaceae (e.g., *K. pneumoniae*, *Enterobacter* spp.) and nonfermenters (i.e., *P. aeruginosa*, *A. baumannii*)
2b	A	Penicillins, cephalothin	TEM-1, TEM-2, TEM-13, SHV-1, SHV-11	Broad-spectrum enzymes inactivated by clavulanic acid	
2be	A	Penicillins, oxyimino-cephalosporins (cefotaxime, ceftazidime, ceftriaxone, cefepime), monobactams	TEM-3, TEM-10, TEM-26, SHV-2, SHV-3, *Klebsiella oxytoca* K1, CTX-M, PER, VEB	Extended broad-spectrum enzymes inactivated by clavulanic acid	
2br	A	Penicillins, resistant to clavulanic acid, tazobactam, sulbactam	TEM-30, TEM-31, SHV-10, SHV-72	Broad-spectrum enzymes with reduced binding to clavulanic acid (inhibitor-resistant TEMs)	
2ber	A	Penicillins, oxyimino-cephalosporins, monobactams, resistant to clavulanic acid, tazobactam, sulbactam	TEM-50, TEM-158	Extended-spectrum enzymes with relative resistance to clavulanic acid	
2c	A	Penicillins, carbenicillin	PSE-1, CARB-3	Carbenicillin-hydrolyzing enzymes inactivated by clavulanic acid	
2ce	A	Carbenicillin, cefepime	RTG-4 (CARB-10)	Extended-spectrum carbenicillinase	
2d	D	Cloxacillin, oxacillin	OXA-1, OXA-2, OXA-10	Cloxacillin-hydrolyzing enzymes with variable inactivation by clavulanic acid	
2de	D	Cloxacillin, oxacillin, oxyimino-cephalosporins, monobactams	OXA-11, OXA-15		
2df	D	Cloxacillin, oxacillin, carbapenems	OXA-23, OXA-51, OXA-58		
2e	A	Cephalosporins	CepA	Cephalosporinases inactivated by clavulanic acid	
2f	A	All *β*-lactams, including carbapenems	KPC, SME, GES, IMI-1	Carbapenem-hydrolyzing non-metallo-*β*-lactamases	
3	B	All *β*-lactams, including carbapenems, with exception of monobactams	IMP, VIM, IND	Metallo-*β*-lactamases	
4	—	Penicillins	Penicillinases from *Burkholderia cepacia*	Penicillinases not inactivated by clavulanic acid	

—: no classification in Ambler system.
